# Supplementary material for: Peptide Adjuvant to Invigorate Cytolytic Activity of NK Cells in an Obese Mouse Cancer Model
Source: Pharmaceutics. 2021 Aug 17;13(8):1279. doi: 10.3390/pharmaceutics13081279 (PMC8401452; doi:10.3390/pharmaceutics13081279)
Supplement: Supplementary file 1 [file pharmaceutics-13-01279-s001.zip › pharmaceutics-1271970-final supp.pdf]

pharmaceutics

MDPI

Supplementary Materials: Peptide Adjuvant to Invigorate Cytolytic Activity of NK Cells in an Obese Mouse Cancer Model

Seungmin Han, Minjin Jung, Angela S. Kim, Daniel Y. Lee, Byung-Hyun Cha, Charles W. Putnam, Kwang Suk Lim, David A. Bull and Young-Wook Won

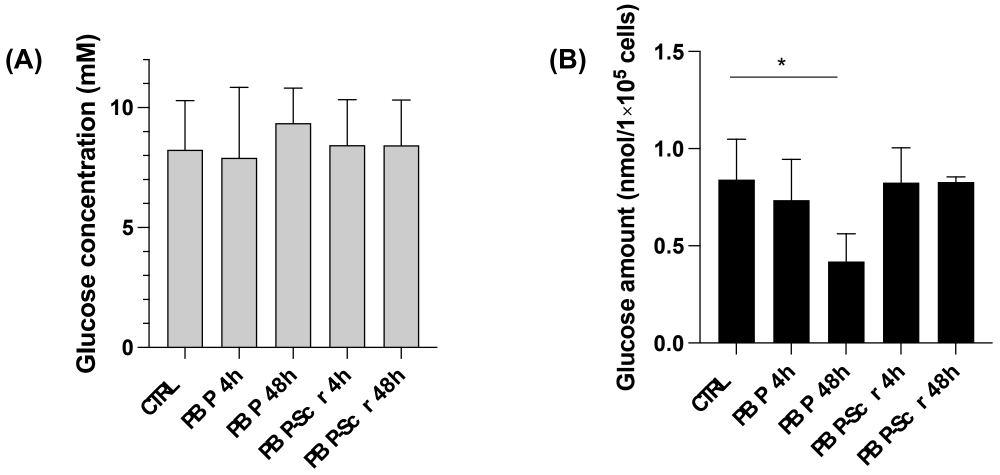

Figure S1. Glucose metabolism changes in B16F10 cell cultures upon PBP or PBP-Scr treatment. (**A**) Glucose concentration in culture medium and (**B**) glucose amount in B16F10 lysates after PBP or PBP-Scr treatment. Hours indicates treatment time. CTRL refers to control (non-treatment) samples. Two-way ANOVA, Tukey test, $p < 0.05$ was used for statistical analysis (* $p < 0.03$).

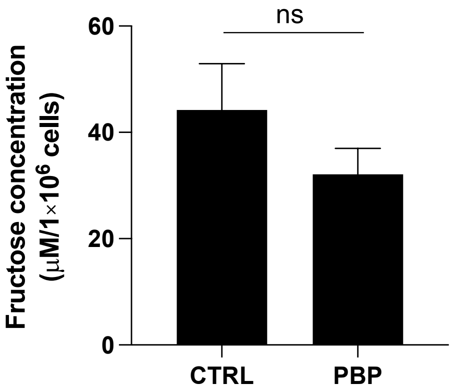

Figure S2. PBP-mediated fructose concentration changes. Fructose concentrations in B16F10 cell lysates after exposure to PBP (150 μM) for 48 hours com-pared to untreated cells. Statistical analysis of the data was analyzed with two-way ANOVA, Tukey test (ns = non-significance).

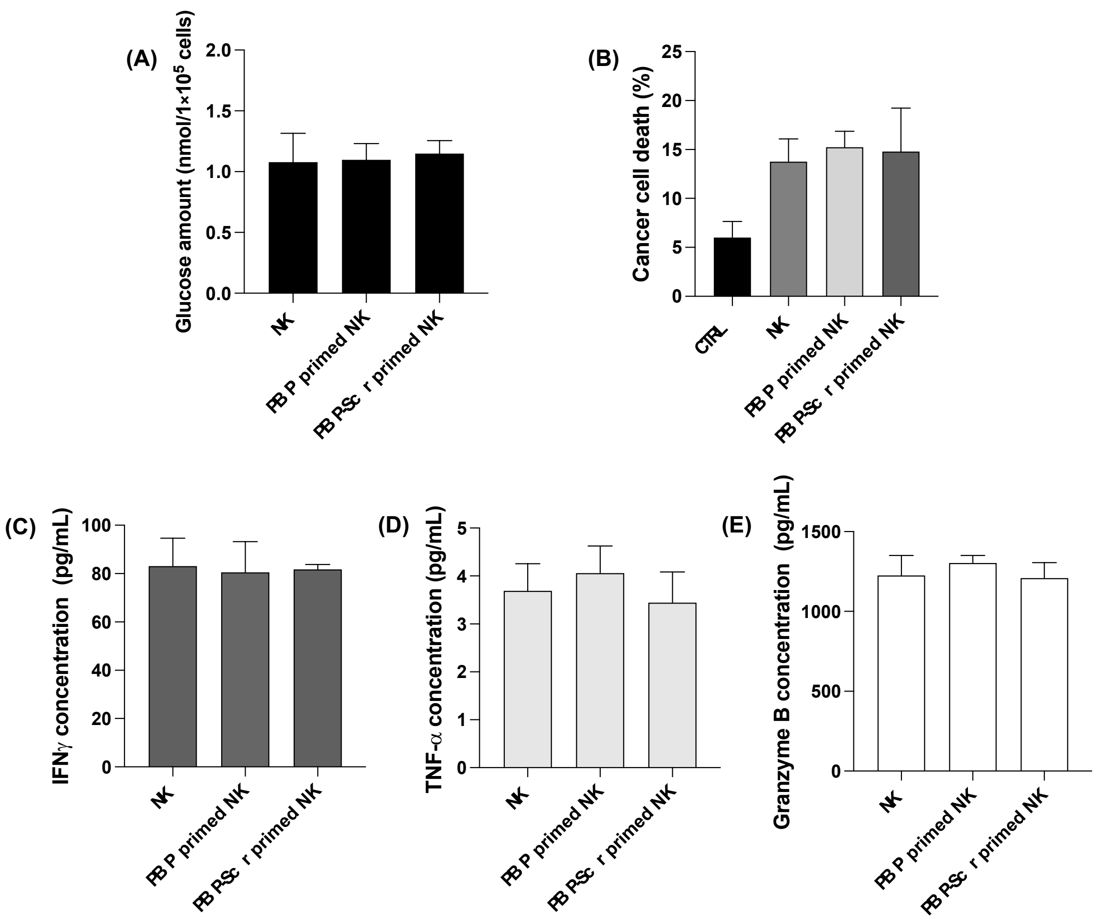

Figure S3. PBP priming effects on NK cells. NK cells were primed by PBP or PBP-Scr for 48 hours. (**A**) Glucose amount in NK cell lysates, (**B**) B16F10 cell death; CTRL data are from B16F10 cells not exposed to NK cells. (**C–E**) Cytokine ELISA of samples harvested from (**B**). There were no statistically significant differences.

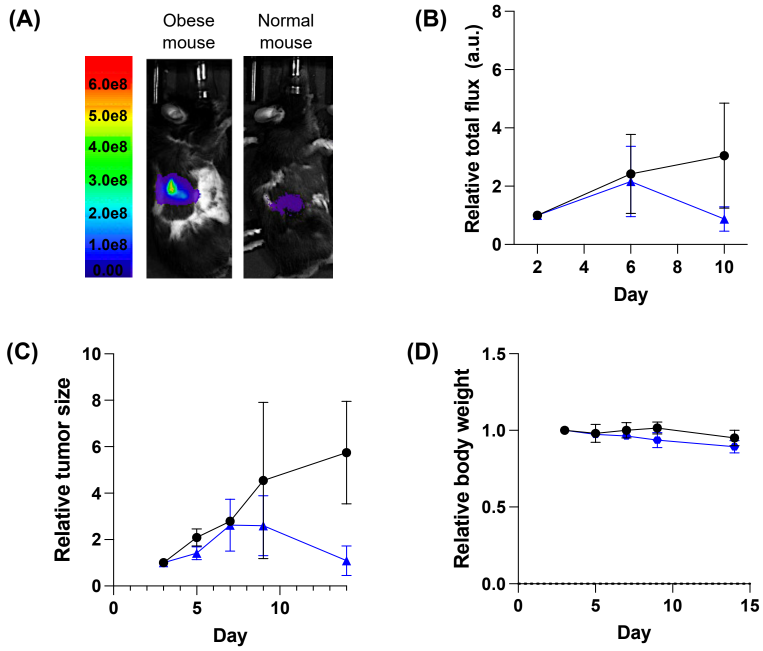

Figure S4. In vivo anti-tumor activity of NK cells in mice bearing B16F10 tumor with obese and normal body weight. (**A**) In vivo luciferase bioluminescence imaging on day 10. (**B**) Relative luciferase flux, and a.u. refers arbitrary unit. (**C**) Relative tumor size based on manual measurements and (**D**) body weight. Black circle/line: NK cells on obese mice; blue circle/line: NK cells on normal body weight mice.

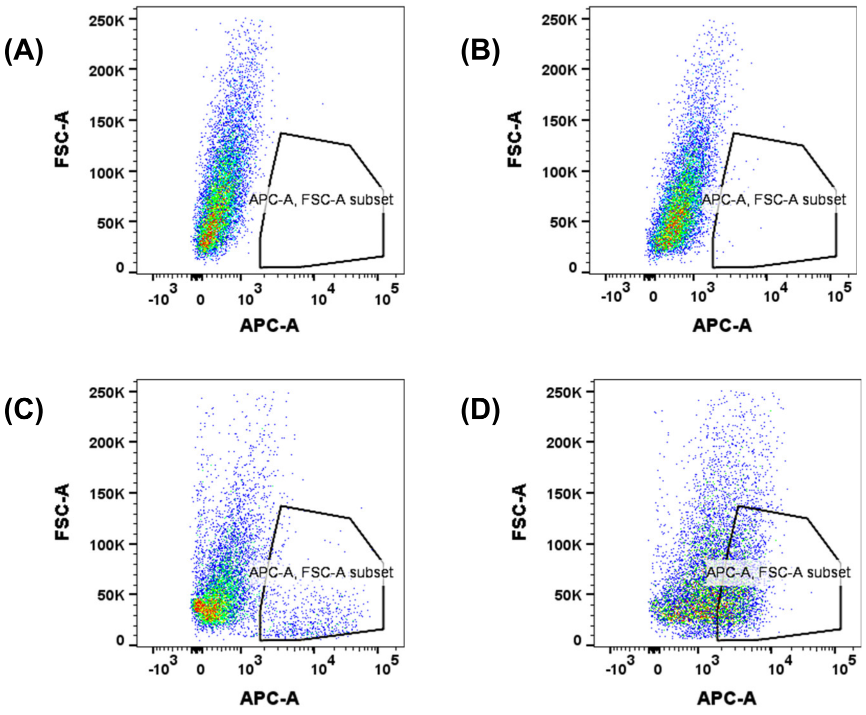

Figure S5. Gating of CD56-APC-stained cells from the total cell population. (**A**) CTRL, (**B**) PBP, (**C**) NK, (**D**) PBP+NK. (**C**) and (**D**) showed rich APC-stained subsets while (**A**) and (**B**) were nearly devoid of APC-stained cells. The reason for different APC intensity between (**C**) and (**D**) is phenotypic changes in NK cells. The APC-stained subset was gated for analysis of CD56 and NKG2A expression on NK cells.

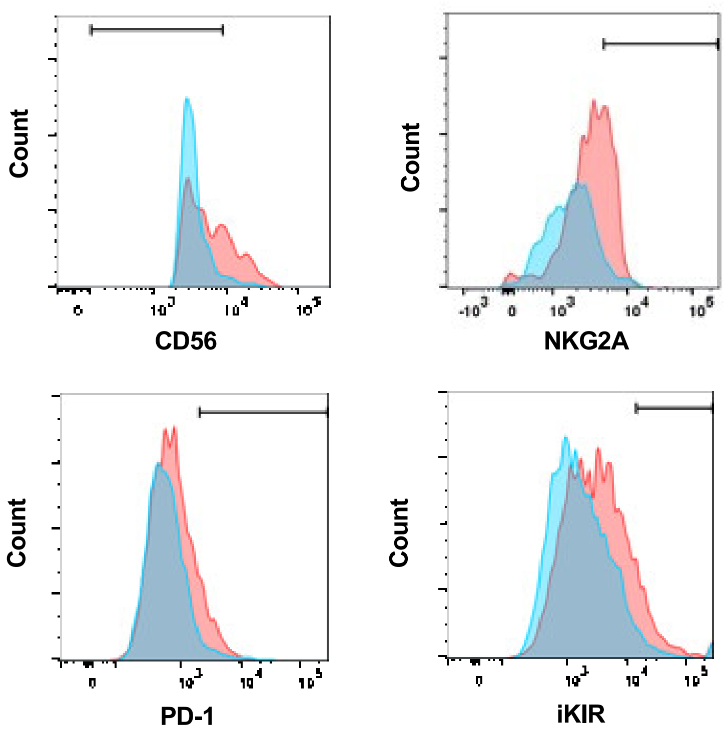

Figure S6. NK cells were analyzed by gating CD56 positive cells from the total cell population. Red histogram: NK group; blue histogram: PBP+NK group. CD56 was divided into CD56bright and CD56dim.

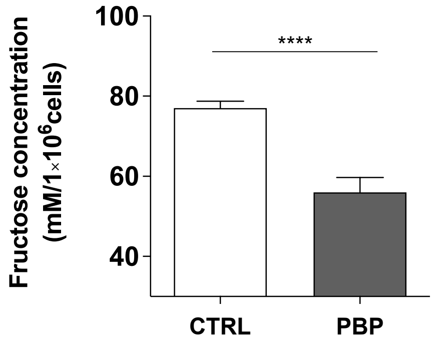

Figure S7. Fructose levels in adipocytes with or without PBP treatment (n = 3). One-way ANOVA, Tukey test, $p < 0.05$ was used for statistical analysis (**** $p < 0.0001$).
